# Importance of monitoring arsenic methylation metabolism in acute promyelocytic leukemia patients receiving the treatment of arsenic trioxide

**DOI:** 10.1186/s40164-021-00205-6

**Published:** 2021-02-06

**Authors:** Yu Zheng, Yuan-Fei Mao, Hui-Jin Zhao, Li Chen, Li-Ning Wang, Yun-Xiang Zhang, Jiong Hu, Jun-Min Li, Xiao-Yang Li, Hong-Ming Zhu

**Affiliations:** Shanghai Institute of Hematology, State Key Laboratory of Medical Genomics, National Research Center for Translational Medicine at Shanghai, Ruijin Hospital Affiliated to Shanghai Jiao Tong University School of Medicine, No. 197 Rui Jin Er Road, Shanghai, 200025 China

**Keywords:** Arsenic trioxide, Arsenic speciation, Arsenic methylation metabolism

## Abstract

**Background:**

Arsenic trioxide [ATO, inorganic arsenite (iAs^III^) in solution] plays an important role in the treatment of acute promyelocytic leukemia (APL). However, the long-term adverse effects (AEs) and the retention of arsenic among APL patients are rarely reported. In this study, we focused on arsenic methylation metabolism and its relationship with chronic hepatic toxicity, as we previously reported, among APL patients who had finished the treatment of ATO.

**Methods:**

A total of 112 de novo APL patients who had completed the ATO-containing treatment were enrolled in the study. Arsenic species [iAs^III^, inorganic arsenate (iAs^V^), and their organic metabolites, monomethylarsonic acid (MMA) and dimethylarsinic acid (DMA)] in patients’ plasma, urine, hair and nails were detected by high-performance liquid chromatography combined with inductively coupled plasma mass spectrometry (HPLC-ICP-MS). Eighteen single nucleotide polymorphisms (SNPs) of the arsenic (+ 3 oxidative state) methylation transferase (*AS3MT*) gene, which was known as the main catalyzer for arsenic methylation, were tested with the polymerase chain reaction method.

**Results:**

The study showed the metabolic pattern of arsenic in APL patients undergoing and after the treatment of ATO, in terms of total arsenic (TAs) and four species of arsenic. TAs decreased to normal after 6 months since cessation of ATO. But the arsenic speciation demonstrated significantly higher portion of iAs^III^ in patient’s urine (40.08% vs. 1.94%, *P* < 0.001), hair (29.25% vs. 13.29%, *P* = 0.002) and nails (30.21% vs. 13.64%, *P* = 0.003) than the healthy controls’, indicating a decreased capacity of arsenic methylation metabolism after the treatment of ATO. Urine primary methylation index (PMI) was significantly lower in patients with both chronic liver dysfunction (0.14 vs. 0.28, *P* = 0.047) and hepatic steatosis (0.19 vs. 0.3, *P* = 0.027), suggesting that insufficient methylation of arsenic might be related to chronic liver disorders. Two SNPs (A9749G and A27215G) of the *AS3MT* gene were associated with impaired urine secondary methylation index (SMI).

**Conclusions:**

The long-term follow-up of arsenic speciation indicated a decreased arsenic methylation metabolism and a probable relationship with chronic hepatic disorders among APL patients after the cessation of ATO. Urine PMI could be a monitoring index for chronic AEs of ATO, and the SNPs of *AS3MT* gene should be considered when determining the dosage of ATO.

## Background

Arsenic trioxide (ATO) has achieved great success in the treatment of acute promyelocytic leukemia (APL) in combination with all-trans retinoic acid (ATRA), improving the rate of complete remission (CR) up to 92% or higher, [1–3] and the long-term overall survival (OS) reaching 87–97% [4–8]. However, the retention and metabolism of ATO in patients are unclear, and the potential long-term arsenic intoxication and secondary carcinogenesis of ATO are rarely reported.

Arsenic speciation by high-performance liquid chromatography and inductively coupled plasma mass spectrometry (HPLC-ICP-MS) has uncovered the pattern of arsenic metabolism recently in the field of toxicology and environmentology. It is now known that the total arsenic (TAs) is composed of inorganic arsenics (iAs) and organic arsenics, and + 3 and + 5 oxidative state inorganic arsenics, i.e. iAs^III^ (as in ATO and As_2_S_3_) and iAs^V^ (as in As_2_O_5_), are the most toxic species [9]. The methylation metabolism of arsenic is considered as the detoxification process [9, 10]. Inorganic arsenics are methylated in the hepatocytes into less toxic metabolites, such as monomethylarsonic acid (MMA) and dimethylarsinic acid (DMA), and excreted via urine. Extra iAs is accumulated in the skin, hair and nails due to the high affinity of iAs^III^ to the sulfydryl (–SH) in the cytokeratin. The urine primary methylation index [PMI = MMA/(iAs^III^ + iAs^V^)] and the secondary methylation index (SMI = DMA/MMA) [11] are recommended to measure the level of arsenic methylation in human body. High urine PMI or SMI represents relatively sufficient metabolism with little iAs retention. Moreover, an *S*-adenosyl methionine dependent arsenic (+ 3 oxidative state) methylation transferase (AS3MT) in the hepatocyte is crucial in the arsenic methylation metabolism [12]. Recent studies also showed that some single nucleotide polymorphisms (SNPs) on the exons or introns of *AS3MT* gene might affect the variations in individual’s ability of arsenic metabolism. The most commonly accepted SNP was the M287T heterozygote on the exon, which was related to better methylation capacity. Other SNPs on the introns (including G12390C, T12590C, T35587C, T14215C, G35991A and so on) were also reported to be associated with elevated DMA or MMA in the urine [13–16].

We have long been focused on the chronic adverse effects (AEs) and retention of ATO in APL patients with ATRA and ATO combination therapy. In the initial report with 5-year follow-up, Hu et al. [1] showed that the concentration of arsenic in patients’ plasma, urine, hair and nails after the treatment decreased significantly compared to patients undergoing ATO treatment, only slightly higher than healthy controls, while no chronic AEs or secondary tumor were observed. Later we reported 15.2% of chronic liver dysfunction and 42.9% of hepatic steatosis (both P < 0.001 compared to healthy controls) in a 12-year follow-up. But the concentration of TAs in patients’ plasma, urine, hair and nails after cessation of ATO were no higher than healthy controls [5]. In an attempt to explore the relationship between chronic hepatic toxicity and the exposure to ATO, in this study we further analyzed the arsenic methylation metabolism by arsenic speciation and its related genes among these patients.

## Methods

### Patients and treatment protocol

Between January 2001 and June 2012, a total of 112 patients with newly diagnosed APL who had completed the whole treatment were retrospectively enrolled in this study, and they were all in persistent molecular remission by the last follow-up. Criteria of diagnosis, cytogenetic analysis and regular reverse transcription-polymerase chain reaction of *PML-RARA* transcripts were performed as previously described [17]. All patients received the ATRA and ATO combination therapy according to the protocol as previously reported, [1, 17] namely ATRA and ATO (plus anthracyclines if the white blood cell count was over 10 × 10^9^/L) in the induction phase, three courses of chemotherapy in the consolidation, and five cycles of ATRA, ATO and low-dose chemotherapy sequential treatment in the maintenance therapy. The dosage of ATO was 5 to 10 mg daily for 28 days per cycle. Patients with persistent remission were treated with ATO for a total of 6 cycles, thus the total dose of ATO for all patients were 840 mg to 1680 mg.

### Arsenic speciation

The blood, urine, hair and nail samples of 112 patients, 112 healthy controls and 7 APL patients undergoing ATO treatment (as positive controls) were collected with informed consent. The study was proved by the Ethics Committee of Ruijin Hospital in Shanghai, China (approval number 2012-48 and 2013-22). Samples of positive controls were collected at 4 time points: before ATO (d0), after 7 days of ATO administration (d7), when achieving CR (usually collected 10–14 days after the induction therapy) and at the end of consolidation therapy (Con, including 3 courses of consolidation therapy with 28 days of ATO in each course). Plasma from the centrifuged blood samples and urine samples were reserved at − 80 °C. Hair samples taken 2 cm near the scalp and fingernails of ten fingers were collected and stored at room temperature shielded from light before testing. All the biological samples were precisely prepared before the test of arsenic speciation as follows:

*Plasma* One tube of the plasma (1–1.5 mL) was mixed with 5 mL of methanol and water mixture (3:1) and extracted overnight on an oscillator twice. All centrifuged supernatant was mixed together and dried with 99.999% nitrogen, then dissolved with 2 mL ultra-pure water.

*Urine* One tube of the urine sample (about 10 mL) was centrifuged before filtered by Sep Pak C18 disposable extraction column (Tianjin Agela Technologies Co., Ltd.) and 0.45 µm filter (Sangon Biotech Co., Ltd.), and diluted for four times.

*Hair and nails* The hair and nail samples were washed according to the “Trace element pollutants washing step” by International Atomic Energy Agency (IAEA) protocol (*Ryabukhin YS, Al-Sharistani H. Activation analysis of hair as an indicator of contamination of man by environmental trace element pollutants. Vienna: International Atomic Energy Agency (IAEA); 1978. Report No.: IAEA/RL/50*), then dried and cut into pieces (< 2 mm). Hair or nail pieces were mixed with 5 mL ultra-pure water and bathed at 90 °C for 6 h for extraction. The samples were centrifuged twice, and the supernatant was reserved for arsenic speciation.

Certified standard material, including AsO_3_^3−^ for iAs^III^, AsO_4_^3−^ for iAs^V^, CH_3_AsO_3_^2−^ for MMA and C_2_H_7_AsO_2_ for DMA, were purchased from Chinese Institute of Metrology (numbers of certification were GBW08666, GBW08667, GBW08668, and GBW08669, respectively). Ultra-pure water (18.2 MΩ cm, NANOpure Diamond, ThermoFisher, USA) was used throughout the test. All the reagents met the requirement of analytical grade.

The high-performance liquid chromatography (ICS-3000, DIONEX, USA) system was combined directly to the inlet of the inductively coupled plasma mass spectrometer (ICP-MS) (X Series II type, Thermo Fisher, USA) for detection of arsenic speciation, including iAs^III^, iAs^V^, MMA and DMA. The mobile phase (20 mM ammonium carbonate) flowed through the anion exchange column at a rate of 1 mL/min for no less than 10 min to obtain pH equilibration (2.0–3.5). The injection volume was 25 µL. With the time of sample injection, DMA, iAs^III^, MMA and iAs^V^ were separated accordingly. Standard compounds containing iAs^III^, iAs^V^, MMA and DMA as mentioned above were used for calibration. The test of arsenic speciation was conducted at the Analysis Center of Tsinghua University, Beijing, China.

### SNPs of*AS3MT* gene

#### DNA extraction

Blood samples were taken from 70 to 112 patients with informed consent of genotyping study. The karyocytes were separated out with red blood cell lysis buffer. The QIAamp DNA mini kit (Qiagen, Chatworth, CA) was used for DNA extraction from the karyocytes.

#### Genotyping

A total of 18 SNPs (T3963C, A4602G, T4740C, A6144T, G7395A, A9749G, A10209G, C12390G, T12590C, T14215C, T14458C, T25986C, T26790C, A27215G, T35587C, A35991G, C37616A, T37950C) were detected with polymerase chain reaction (PCR) method [13] (see Table 1 for the primer sequences of each genotype). Amplification was performed in a 25 µL reaction mixture containing 0.2 µL GoTaq (Promega, Madison, WI, USA), 5 µL 5× Colorless GoTaq Reaction Buffer, 2 µL dNTPs (2.5 mM), 1 µL primer (10 µM) and 2 µL DNA sample (30–100 ng/µL). The PCR protocol consisted of initial denaturation at 96 °C for 2 min, followed by 35 cycles of denaturation at 95 °C for 30 s, annealing at 60 °C for 30 s, and extension at 72 °C for 30 s, and a final extension at 72 °C for 5 min. The PCR products were then sent to Shanghai SimpleGene Clinical Laboratory for Sanger sequencing.

**Table 1:** SNPs and primer sequence for PCR-based genotyping

No.	Gene	Primer	Sequence	PCR size (bp)
1	3963 (T to C)	3963-F	AATGAATAGAAATGATCGTTACAAG	377
		3963-R	ATGGAACACTTCACGAATTTGTATG	
2	4602 (A to G)	4602-F	CGAAGAAACTTGTGGGCCAGA	260
		4602-R	TCGCTCCACTGCGATTTTCAC	
3	4740 (T to C)	4740-F	CGAAGAAACTTGTGGGCCAGA	223
		4740-R	CTGATTTAAATGAACACTCACCT	
4	6144 (A to T)	6144-F	GGTCACTAGGGAATTAACCCG	414
		6144-R	CCAAGGTTGATTAGTGGGTGC	
5	7395 (G to A)	7395-F	CGCCTATGGGACAGAAACCTT	154
		7395-R	CTAAGGGACAGAGTGAGACTC	
6	9749 (A to G)	9749-F	CCAGACCAGCTTGAACAACATGGC	388
		9749-R	CCCCAAGATCAGAGACAGAGT	
7	10209 (A to G)	10209-F	AAAGAGAGGAGGGAGGCGGTA	291
		10209-R	GAGCTCTTAGTTCAAGGGGGA	
8	12390 (C to G)	12390-F	TTTAGGAGACAGACACTCTTAGAATG	321
		12390-R	ACTTTGTGTGTCCTGATTTCTTCTG	
9	12590 (T to C)	12590-F	GTTTCAGCATGGTGGGGAGTT	172
		12590-R	AGCCTCATCCTGGCTATTAGC	
10	14215 (T to C)	14215-F	CTGTACAATGGTAACCCCCCA	101
		14215-R	GCATGTCATCAGTTATCTTTCT	
11	14458 (T to C)	14458-F	GTGCTGGAGATGAACCGTGAA	232
		14458-R	GCAAGGGCAAGAGCAGAAAGA	
12	25986 (T to C)	25986-F	CTTCTGGGCATTTTTTCTTCTA	236
		25986-R	GTTCTCTCAGGTAGTGAAAGCC	
13	26790 (T to C)	26790-F	TGATCGCCTGACATTCCTGGT	203
		26790-R	CCAAGTGTCTCCAATTGTCCTG	
14	27215 (A to G)	27215-F	GCCTATCTGGCCAAACTCTTG	221
		27215-R	CATGTTACCCAGGCTGGTGTC	
15	35587 (T to C)	35587-F	CAGCAGTCTTGTCTTTTAAAT	188
		35587-R	GCTAAAACGACTTCTCTTCCC	
16	35991 (A to G)	35991-F	CACGTGCAAATGACAACCCCA	227
		35991-R	GTTTGATTTAGGTTGACTTACA	
17	37616 (C to A)	37616-F	GCCAAATCATGTTTGGTAGGAGG	370
		37616-R	GTGCAGTGGTGCAATCATAGC	
18	37950 (T to C)	37950-F	CATGGTGAGACCCCCATCTCT	462
		37950-R	CCAGCTCCTGATGATAATGACC	

### Statistical analysis

The concentration of TAs and arsenic speciation were documented in the form of “mean ± standard deviation (SD)”, as well as the percentage of different species of arsenic, and compared between groups by Student’s *t* test. While PMI and SMI were shown as “median (minimum–maximum)” because of non-normal distribution of the data, and compared between groups by Kolmogorov–Smirnov test. *P* < 0.05 was determined for statistically significant difference. All statistical analyses were performed using SPSS 20.0 statistical package.

## Results

**Fig. 1:**
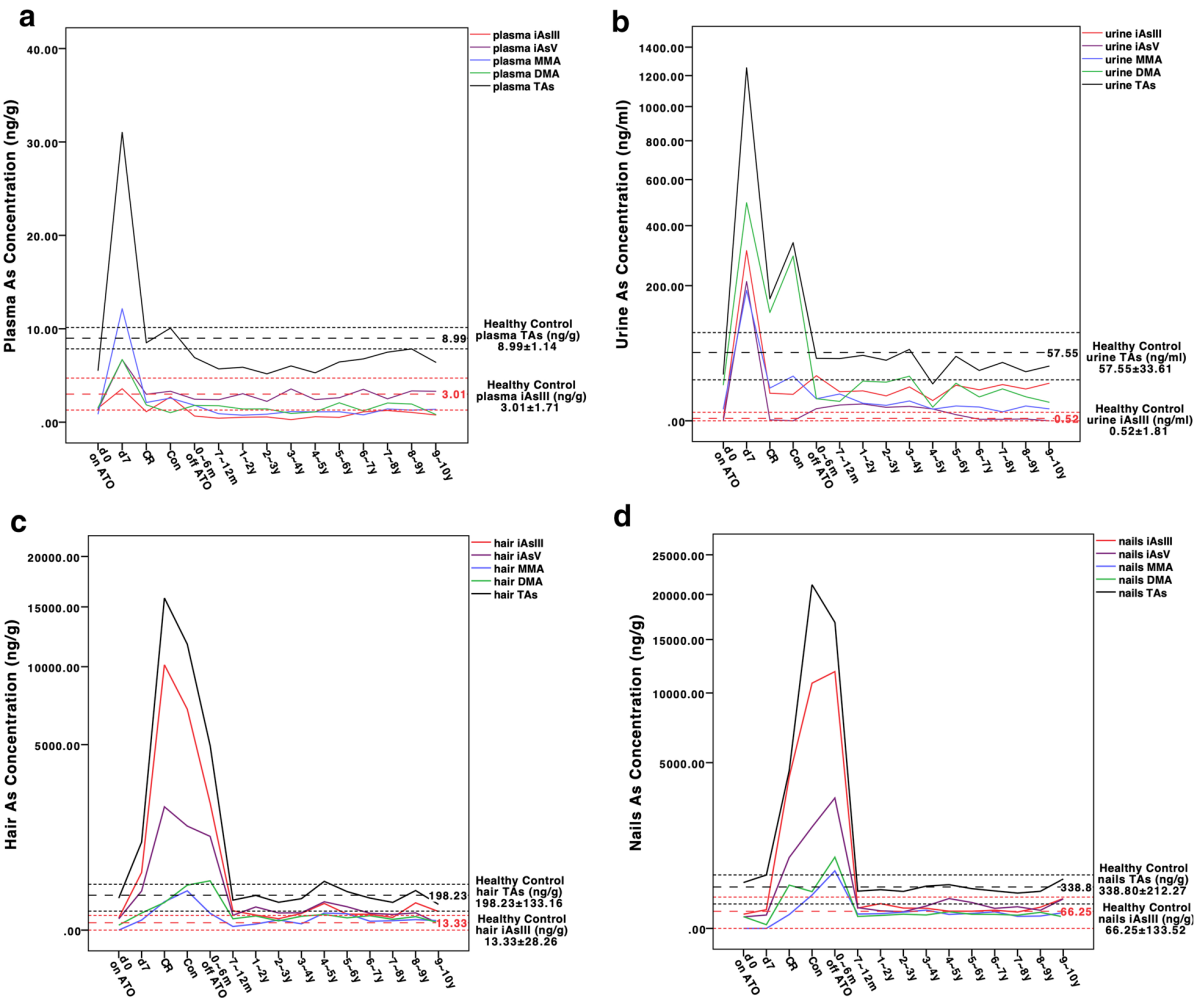
TAs and arsenic speciation by time on and off ATO in patients’ plasma (**a**), urine (**b**), hair (**c**) and nails (**d**). TAs and arsenic speciation were pointed at mean value for each group. The reference lines in black and red were mean ± Standard Deviation (SD) for TAs and iAs^III^ of the healthy controls, respectively. TAs: total arsenic; ATO: arsenic trioxide; iAs^III^: inorganic arsenite

The 112 patients were divided into different groups according to their time intervals between the cessation of ATO and the arsenic test, namely off ATO 0–6 months (0–6 months, 8 cases), 7–2 months (10 cases), 1 –2 years (17 cases), 2 –3 years (12 cases), 3 –4 years (6 cases), 4 –5 years (6 cases), 5 –6 years (13 cases), 6 –7 years (10 cases), 7 –8 years (14 cases), 8–9 years (13 cases) till 9–10 years (3 cases). Seven positive controls were tested at 4 time points: d0, d7, CR and Con. The trend of TAs accumulation and excretion in plasma, urine, hair and nails had been previously reported (black curves in Fig. 1) [5]. To be brief, TAs in plasma and urine were excreted quickly to normal levels within 0 to 6 months, no higher than that of the healthy controls, but in hair and nails the excretion was delayed till after 6 months off ATO. Therefore, when comparing the long-term TAs and arsenic speciation in hair and nails, patients who had finished the ATO treatment for less than 6 months were excluded from the calculation.

## Arsenic speciation

The trend of average concentration of iAs^III^, iAs^V^, MMA and DMA in patients’ plasma, urine, hair and nails is also shown in Fig. 1. When patients were undergoing ATO treatment, arsenic speciation in their plasma demonstrated that the main component of TAs was organic arsenic MMA and DMA, but not iAs^III^ as in ATO (Fig. 1a), indicating that the methylation capacity of patients was sufficient to metabolize ATO during continuous infusion, and excessive iAs^III^ was quickly excreted through the urine since iAs^III^ was the second highest component of TAs in the urine during ATO treatment, second only to DMA (Fig. 1b). Therefore, the concentration of iAs^III^ was relatively low in patients’ blood, whereas higher in their urine. Meanwhile, due to high affinity of -SH in hair and nails to iAs^III^, it was the main component of TAs in patients’ hair and nails (Fig. 1c, d) during the treatment, and decreased till 6 months off ATO, which was consistent with the trend of TAs in hair and nails.

**Fig. 2:**
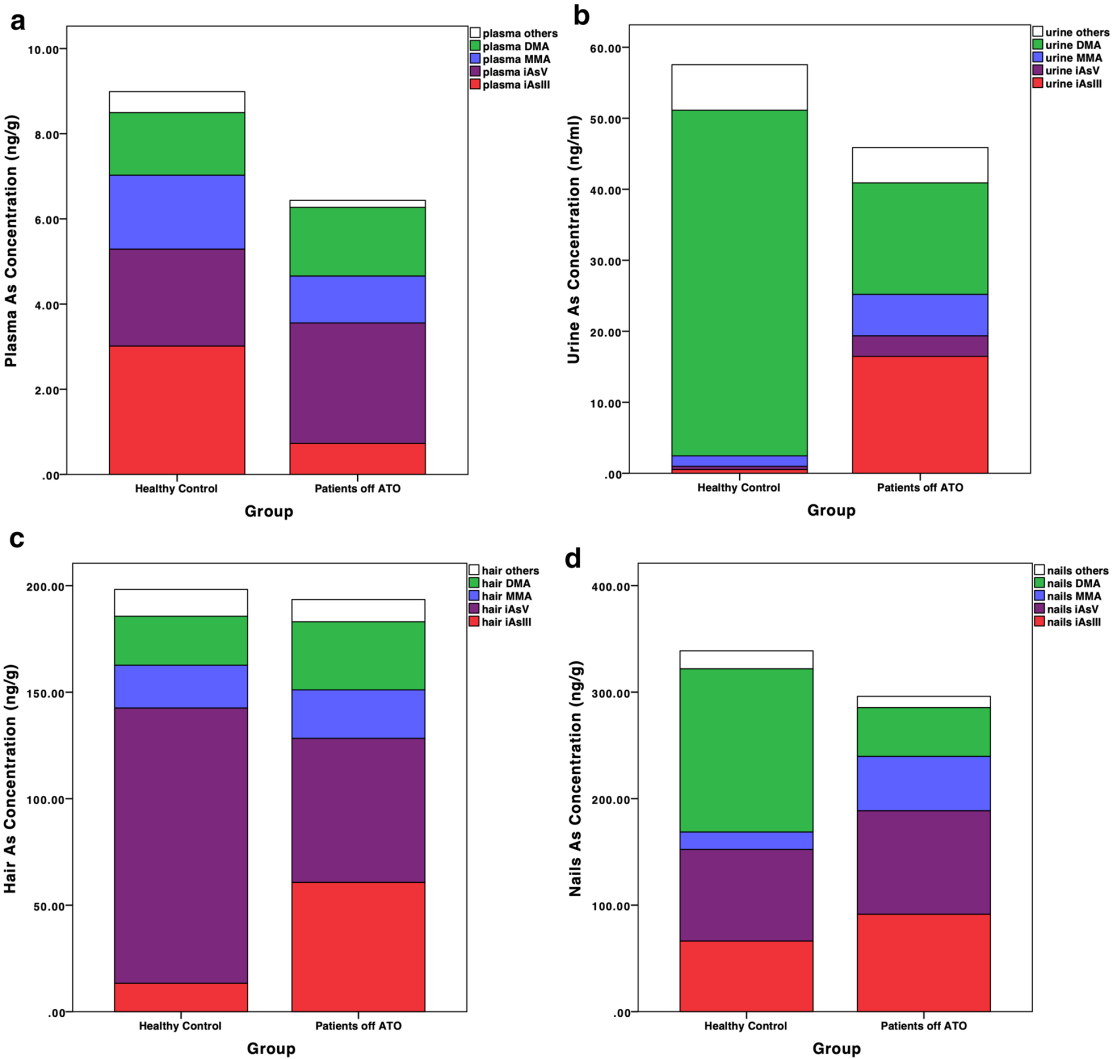
Arsenic speciation in plasma (**a**), urine (**b**), hair (**c**) and nails (**d**) of patients off ATO compared to healthy controls. *ATO* arsenic trioxide

However, long-term follow-up showed that the percentage of iAs^III^ in patients’ urine, hair and nails were still significantly higher than the healthy controls, with an average of 40.08 ± 28.90% vs. 1.94 ± 7.19% (*P* < 0.001) in urine, 29.25 ± 16.44% vs. 13.29 ± 26.54% (*P* = 0.002) in hair, and 30.21 ± 16.70% vs. 13.64 ± 30.55% in nails (*P* = 0.003) (Table 2). The patterns of arsenic composition in patients and healthy controls were illustrated more specifically in Fig. 2. Obviously in urine samples (Fig. 2b) it was the most significantly different, with organic arsenic reaching over 80% (78.92% of DMA and 2.49% of MMA) in the healthy controls, while iAs counting nearly a half (40.08% of iAs^III^ and 8.72% of iAs^V^) in the patients off ATO (Table 2). Since the recent intake of iAs, which was reflected by the sum of iAs^III^ and iAs^V^ in plasma (Fig. 2a), was no higher in patients, lower percentage of organic arsenic in the urine indicated insufficient arsenic methylation metabolism among patients.

**Table 2:** TAs and arsenic speciation of patients and healthy control

	TAs (ng/g)	iAs^III^%	iAs^V^%	MMA%	DMA%	PMI	SMI
Plasma							
Patients	6.43 ± 1.56	11.40 ± 9.98	44.47 ± 19.81	17.19 ± 13.39	24.50 ± 15.47	0.25 (0–5.04)	1.75 (0.06–37.14)
Control	8.99 ± 1.14	33.87 ± 18.92	25.26 ± 16.56	19.29 ± 14.53	16.13 ± 13.03	0.28 (0–8.74)	0.82 (0–19.2)
*P* value	< 0.001	< 0.001	< 0.001	0.28	< 0.001	0.742	0.002
Urine							
Patients	45.28 ± 33.28	40.08 ± 28.90	8.72 ± 11.42	15.64 ± 14.05	26.36 ± 26.64	0.27 (0–5.52)	1.21 (0–143.01)
Control	57.55 ± 33.61	1.94 ± 7.19	1.37 ± 6.31	2.49 ± 3.52	78.92 ± 18.79	0.27 (0–1.24)	16.36 (3.32–120.34)
*P* value	0.009	< 0.001	< 0.001	< 0.001	< 0.001	0.001	< 0.001
Hair							
Patients	195.43 ± 202.25	29.25 ± 16.44	34.26 ± 20.82	12.05 ± 12.95	18.87 ± 11.95	0.13 (0–2.16)	1.35 (0–91.46)
Control	198.23 ± 133.16	13.29 ± 26.54	53.00 ± 48.00	14.93 ± 35.01	12.57 ± 26.58	0 (0–0.21)	0 (0–0.01)
*P* value	0.96	0.002	0.012	0.558	0.132	0.001	0.007
Nails							
Patients	294.65 ± 158.94	30.21 ± 16.70	32.85 ± 18.08	17.70 ± 12.08	15.72 ± 10.14	0.24 (0–2.64)	0.84 (0–9)
Control	338.80 ± 212.27	13.64 ± 30.55	20.07 ± 35.56	8.71 ± 26.27	52.57 ± 44.26	0 (0–0.45)	0.35 (0–0.69)
*P* value	0.353	0.003	0.034	0.032	< 0.001	0.002	0.514

TAs: total arsenic; iAs^III^%: inorganic arsenite/TAs × 100%; iAs^V^%: inorganic arsenate/TAs × 100%; MMA%: monomethylarsonic acid/TAs × 100%; DMA%: dimethylarsinic acid/TAs × 100%; PMI: primary methylation index; SMI: secondary methylation index

**Fig. 3:**
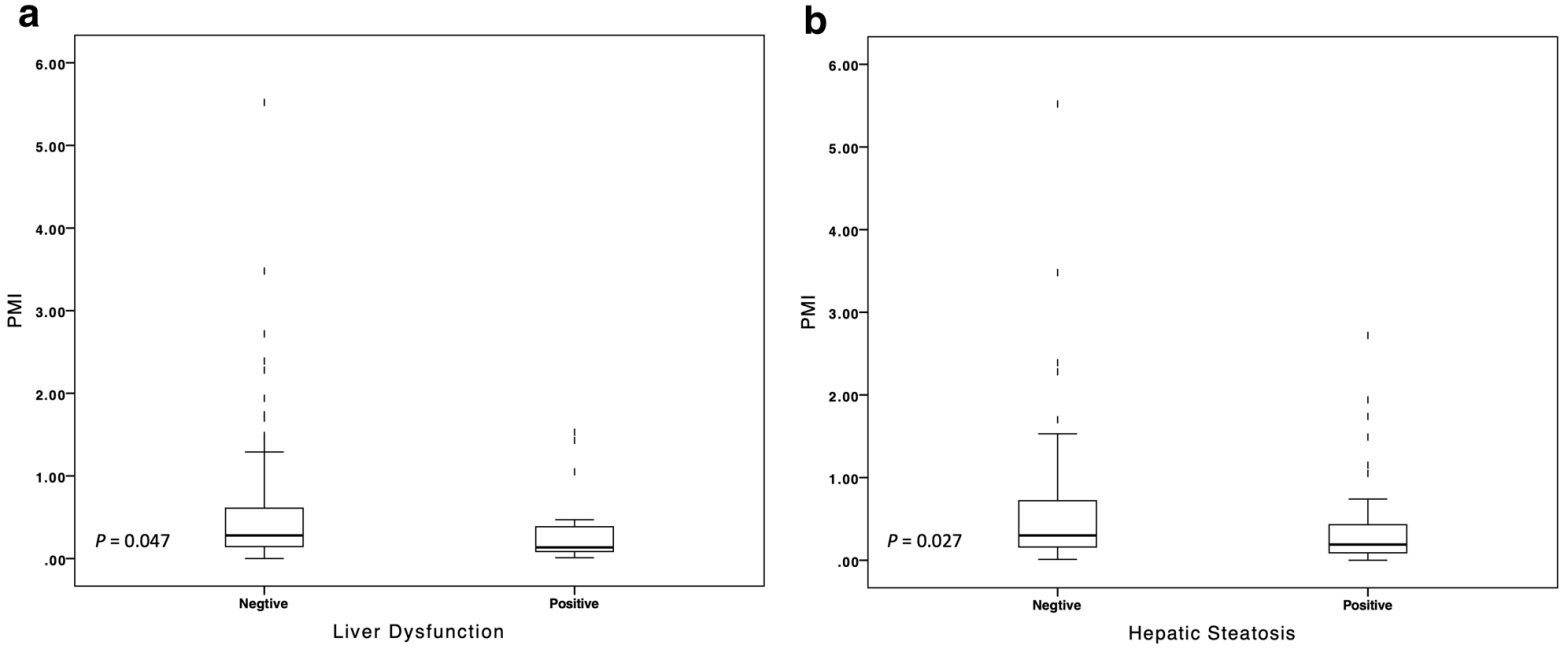
The relationship of urine PMI with chronic liver dysfunction (**a**) and hepatic steatosis (**b**). *PMI* primary methylation index

Since liver dysfunction and hepatic steatosis were the main chronic AEs in the follow-up, we further evaluate the relationship between liver disorders and urine arsenic metabolism. Although the percentage of different species of arsenic was of no significant difference between patients with and without liver disorders, the urine PMI was lower in patients with liver dysfunction (0.37 vs. 0.59, *P* = 0.047) and hepatic steatosis (0.40 vs. 0.68, *P* = 0.027) compared to patients with normal liver examination (Fig. 3). It suggested that lower capacity of transforming iAs into MMA in patients might associate with more iAs accumulation in the long run and the consequently chronic liver toxicity.

### SNPs of *AS3MT* gene

The 18 SNPs of *AS3MT* gene were genotyped among 70 patients. The concentration of urine TAs, the percentage of arsenic speciation, PMI and SMI were compared between the wild type and the mutation groups in each SNP. The data showed that two SNPs were associated with elevated urine SMI among patients (Table 3) when both mutated from allele A to G, with SMI 4.2 vs. 12.0 (*P* = 0.011) for A9749G, and 5.9 vs. 12.6 for A27215G (*P* = 0.017), indicating more sufficient methylation metabolism of arsenic. However, none of the 18 SNPs affected the urine PMI or chronic liver disorders according to the multivariant linear regression or logistic regression.

**Table 3:** SNPs of *AS3MT *and the urine arsenic methylation metabolism

SNPs^a^	TAs (ng/mL)	iAs^III^%	iAs^V^%	MMA%	DMA%	PMI	SMI
9749							
AA	43.66 ± 35.79	41.16 ± 31.22	10.55 ± 13.83	16.54 ± 13.44	23.39 ± 24.27	0.21 (0.02–2.39)	1.05 (0–41.17)
AG & GG	41.61 ± 30.20	29.04 ± 30.13	5.54 ± 6.39	5.96 ± 3.64	46.45 ± 33.32	0.21 (0.02–1.33)	7.5 (0–29.06)
*P* value	0.874	0.294	0.296	0.024	0.02	0.989	0.011
27215							
AA	41.66 ± 30.47	43.23 ± 30.00	9.09 ± 12.62	15.69 ± 13.04	23.11 ± 23.08	0.20 (0.02–2.39)	0.96 (0–143.01)
AG & GG	54.73 ± 39.34	23.73 ± 27.78	6.29 ± 6.38	9.42 ± 14.73	41.65 ± 34.93	0.26 (0.02–1.53)	6.41 (0–41.17)
*P* value	0.209	0.044	0.458	0.147	0.027	0.71	0.017

SNP: single nucleotide polymorphism; *AS3MT*: (+ 3 oxidative state) methylation transferase; TAs: total arsenic; iAs^III^%: inorganic arsenite/TAs × 100%; iAs^V^%: inorganic arsenate/TAs × 100%; MMA%: monomethylarsonic acid/TAs × 100%; DMA%: dimethylarsinic acid/TAs × 100%. PMI: primary methylation index; SMI: secondary methylation index

^a^All the other SNPs of AS3MT showed no significant influence on arsenic metabolism, therefore only A9749G and A27215G are presented

## Discussion

The ATRA and ATO combination therapy for APL has brought significant advantages in both laboratory studies and clinical settings [1], especially when compared to the ATRA and anthracycline-based chemotherapy [3, 7, 8], suggesting that the addition of arsenic in the front-line therapy would guarantee a longer remission duration and survival. However, ATO was still considered as a potential toxicant as well as a carcinogen, which was commonly observed after long-time exposure to environmental contamination [18, 19]. In our previous study, the incidence of mild liver dysfunction (15.2%) and hepatic steatosis (42.9%) in patients was significantly higher compared to the healthy controls [5].

Toxicological studies have proved that the plasma and urine arsenic is related to the short-term arsenic exposure, while arsenic in hair and nails represents the long-term retention of iAs [10]. Urine arsenic speciation reveals the methylation capacity, with the least toxic DMA accounting for 60–80% of urine TAs. In our 5-year follow-up, Hu et al. [1] reported no significant TAs retention in patients’ plasma, urine, hair and nails. While in this study the concentration of TAs in patients’ plasma and urine was even lower than healthy controls (Table 2), indicating less arsenic exposure or intake from patients’ recent environment. When arsenic speciation was taken into consideration, the metabolism, excretion and accumulation of ATO in patients’ plasma, urine, hair and nails was profiled with more details. We reported a quick metabolism and excretion of iAs^III^ in patients’ plasma and urine right after the administration of ATO, while the accumulation of iAs^III^ in their hair and nails continued until 6 months after the cessation of ATO.

More importantly, the urine arsenic speciation revealed significantly different capacity of arsenic methylation between patients and healthy controls. The urine arsenic speciation in healthy controls was basically in line with the normal pattern of arsenic metabolism and distribution, with DMA reaching 78.92% of the urine TAs. On the contrary, the iAs^III^% in patients’ urine was significantly higher. The high level of unmethylated iAs^III^ might accumulate in -SH enriched tissues and organs, which may be the cause of higher percentage of iAs^III^ in patients’ hair and nails even after 6 months off ATO, as well as ATO-associated chronic AEs. The impact of arsenic exposure on the methylation capacity was still not clear, but recent studies suggested that change of methylation capacity is dependent on the exposure time, concentration and the duration after arsenic exposure. Xu et al. [20] analyzed urine arsenic of patients with sub-acute arseniasis, chronic high-dose exposure and the controls, and reported that the sub-acute arseniasis group had significantly impaired methylation metabolism than the chronic high-dose group. Wei et al. [21] concluded that the arsenic methylation efficiency was significantly lower due to chronic exposure to high levels of arsenic in the environment. But Huang et al. [22] performed arsenic speciation for non-tumor residents in southern Taiwan with a 15-year interval after cessation of arsenic ingestion, and demonstrated an increase of methylation. In our study, the decreased capacity of methylation metabolism in patients seemed to continue even after 10 years off ATO.

The urine primary and secondary methylation indexes (PMI and SMI) simply reflect the methylation level of arsenic in human body. Studies have shown that lower urinary arsenic PMI or SMI might increase the risk of skin lesions, hypertension [18] and bladder cancer [23]. In our study, we showed that urine PMI was significantly lower in patients with liver dysfunction and hepatic steatosis, indicating that lower methylation capacity was associated with the incidence of chronic liver disorders. As we know, ATO-related acute liver dysfunction during the treatment of APL is common, with the incidence between 33 and 75% [1, 24]. Lo-Coco et al. [3] reported that the incidence of grade 3–4 liver dysfunction in the ATRA + ATO group was even up to 57%. When ATO is binding to the -SH, it will produce more intracellular reactive oxygen species and lead to cytochrome C leakage, DNA damage and apoptosis [25]. More importantly, acute liver dysfunction might lead to a decrease in the capacity of arsenic methylation [26], thus relatively higher iAs^III^% in the urine, hair and nails would last for a long time as shown in our study. Incomplete methylation of iAs^III^ would cause tissue and organ damage under oxidative stress, including chronic liver dysfunction and hepatic steatosis as presented in our previous study. Santra et al. [27] reported that the fatty infiltration of liver in mice feeding with iAs was not observed until the 12th month, and liver fibrosis till the 15th month. Mazumder et al. [28] underwent liver biopsy in 69 patients in India diagnosed as arseniasis due to polluted drinking-water, and 63 of them presented non-cirrhosis liver fibrosis, indicating that the acute apoptosis of hepatocytes was likely to develop into chronic liver dysfunction manifested as fatty infiltration and fibrosis. Though intravenously administrated ATO might also cause chronic liver disorders in a similar way, there was no liver fibrosis documented yet in our follow-up study. Of note, almost a half of our patients enrolled in the follow-up study had hepatic steatosis, which might affect the capacity of arsenic methylation. So continuous monitoring of urine PMI during and after the treatment of ATO may predict the risk of chronic AEs caused by ATO. Besides, we should be cautious that these patients with hepatic steatosis might develop into non-cirrhosis liver fibrosis in case of further arsenic exposure, thus long-term clinical observation is warranted.

In 2001 Lin et al. [12] discovered an *S*-adenosyl methionine (SAM) dependent arsenic (+ 3 oxidative state) methylation transferase (AS3MT) in the hepatocyte. Since then, a series studies have proved the importance of AS3MT in the arsenic methylation metabolism. The transformation of iAs^III^ into MMA and DMA is mainly catalyzed by AS3MT. AS3MT polymorphism has been reported in several studies. Different genotypes of *AS3MT* gene produced different phenotypes of AS3MT enzymes, leading to variation in the methylation capacity and the metabolites of urine arsenic. The research into the genotyping for the original inhabitants with or without arsenic contamination showed that inhabitants living in the highly contaminated areas for thousand years tended to express more powerful genotypes of *AS3MT* with stronger methylation capacity [29]. Some SNPs on the exons or introns of *AS3MT* gene were related to individual’s differences in the ability of arsenic metabolism. The best clarified was that the M287T heterozygote on the exons, which was associated with increased activity of AS3MT enzymes, more organic arsenic in the urine and higher methylation capacity [13]. However in Asian population, the M287T mutation was fairly rare [30]. In our study, the M287T heterozygote (T14458C) were detected only in 3 out of 70 patients, with no significant influence on the arsenic metabolism as measured by arsenic speciation in patients’ urine. Other SNPs including G7395A, G12390C, G12390T, T35587C, T14215C, G35991A, which were considered to have more sufficient arsenic methylation in the urine [31, 32], were also not noticed in this study. However, both A9749G and A27215G showed significant influence on the urine SMI, indicating a more sufficient arsenic methylation metabolism. It may help explain the variant incidence of acute AEs when patients are treated with ATO, which was also reported in Lu et al.’s study [15] that patients with both 35991 (rs10748835) AA and 35587 (rs11191453) TC + CC genotypes had the highest DMA% and SMI, but the lowest iAs%, serum alanine aminotransferase and aspartate aminotransferase level, indicating that additive effects exist on arsenic metabolism and liver function. Nevertheless, none of the 18 SNPs was related to decreased PMI and chronic liver disorders in our study. Other SNPs or genes should be explored to better understand the changes in arsenic methylation capacity and the pathological process of chronic AEs among APL patients.

## Conclusions

In summary, ATO was generally safe with no obvious TAs retention for patients with APL, but the long-term follow-up of arsenic speciation revealed an impaired methylation capacity with higher iAs% in their urine, hair and nails after the cessation of ATO treatment, which might be associated with the occurrence of chronic liver disorders, as indicated by lower urine PMI. Two SNPs (A9749G and A27215G) of the *AS3MT* gene might be responsible for a more sufficient arsenic methylation metabolism with higher urine SMI. Urine PMI could be a monitoring index for chronic AEs of ATO, and the SNPs of *AS3MT* gene should be considered when determining the treatment dosage of ATO. The mechanisms for impaired capacity of arsenic methylation and chronic liver disorders are yet to be discovered.

## Data Availability

The datasets used and analyzed during the current study are available from the corresponding author on reasonable request.

## Abbreviations

ATO: Arsenic trioxide
APL: Acute promyelocytic leukemia
ATRA: All-trans retinoic acid
CR: Complete remission
OS: Overall survival
TAs: Total arsenic
iAs: Inorganic arsenics
MMA: Monomethylarsonic acid
DMA: Dimethylarsinic acid
AS3MT: Arsenic (+3 oxidative state) methylation transferase
SNPs: Single nucleotide polymorphisms
AEs: Adverse effects
PCR: Polymerase chain reaction
PMI: Primary methylation index
SMI: Secondary methylation index
